# Protein Kinase Regulated by dsRNA Downregulates the Interferon Production in Dengue Virus- and dsRNA-Stimulated Human Lung Epithelial Cells

**DOI:** 10.1371/journal.pone.0055108

**Published:** 2013-01-25

**Authors:** Yuye Li, Jiong Xie, Siyu Wu, Jun Xia, Peifen Zhang, Chao Liu, Ping Zhang, Xi Huang

**Affiliations:** 1 Department of Immunology, Institute of Human Virology, Zhongshan School of Medicine, Sun Yat-sen University, Guangzhou, China; 2 Key Laboratory of Tropical Diseases Control (Sun Yat-sen University), Ministry of Education, Guangzhou, China; Institut Pasteur, France

## Abstract

**Background:**

Dengue virus (DENV) is found in the tropical and subtropical regions and affects millions of people annually. Currently, no specific vaccine or antiviral treatment against dengue virus is available. Innate immunity has been shown to be important for host resistance to DENV infection. Although protein kinase regulated by double-stranded RNA (PKR) has been found to promote the innate signaling in response to infection by several viruses, its role in the innate response to DENV infection is still unclear. Our study aimed to investigate the role of PKR in DENV-induced innate immune responses.

**Methodology/Principal Findings:**

By RNAi, silencing of PKR significantly enhanced the expression of interferon (IFN)-β in DENV infected human lung epithelial A549 cells. Western blot and immunofluorescence microscopy data showed that PKR knockdown upregulated the activation of innate signaling cascades including p38 and JNK mitogen-activated protein kinases (MAPKs), interferon regulatory factor-3 and NF-κB, following DENV2 infection. Likewise, a negative regulatory effect of PKR on the IFN production was also observed in poly(IC) challenged cells. Moreover, the PKR knockdown-mediated IFN induction was attenuated by RIG-I or IPS-1 silencing. Finally, overexpression of a catalytically inactive PKR mutant (K296R), but not of a mutant lacking dsRNA binding activity (K64E) or the double mutant (K64EK296R), reversed the IFN induction mediated by PKR knockdown, suggesting that the dsRNA binding activity is required for PKR to downregulate IFN production.

**Conclusions/Significance:**

PKR acts as a negative regulator of IFN induction triggered by DENVs and poly(IC), and this regulation relies on its dsRNA binding activity. These findings reveal a novel regulatory role for PKR in innate immunity, suggesting that PKR might be a promising target for anti-DENV treatments.

## Introduction

Dengue virus (DENV), a member of the *Flaviviridae* family, is one of the most prevalent arthropod-borne viruses. DENVs are enveloped positive single-stranded RNA viruses, comprising four distinct serotypes (DENV1–4) [1], [2]. Primary infection with these four serotypes only causes a mild self-limiting illness, termed dengue fever (DF), but subsequent infection with other DENV serotypes then often causes more severe life-threatening diseases, including dengue hemorrhagic fever (DHF) and dengue shock syndrome (DSS) [3]. DENVs are found in the tropical and subtropical regions and affect 50–100 million people annually, among which 1% are hospitalized [4], [5]. Despite its clinical significance, no specific vaccine or antiviral treatment against dengue virus is currently available.

The innate immune response is the first line of host defense against viruses, and in particular, interferons (IFNs) can directly determine the different clinical outcomes [6]. Experimentally, DENVs are susceptible to IFN treatment *in vivo* and *in vitro*. In murine models, animals with deficits in IFN signaling had a higher mortality following DENV infections than wild-type mice [7]. In cultured cell lines, pretreatment with IFNs is sufficient to prevent DENV replication [8]. Therefore, a more complete understanding of the precise anti-DENV innate immune signaling pathway, including the roles of both positive and negative regulators, could help in the development of novel anti-DENV vaccines or drugs.

Double-stranded RNA (dsRNA), a pathogen-associated molecular pattern (PAMP), is an intermediate product in the life cycle of both RNA viruses and DNA viruses [9]. Extracellular or cytoplasmic dsRNA is sensed by two different groups of cellular pattern recognition receptors (PRRs), namely membrane-bound Toll-like receptor 3 (TLR3) and cytoplasmic retinoic acid-inducible gene I (RIG-I)-like helicases (RLHs), including RIG-I and melanoma differentiation-associated gene 5 (MDA-5) [10]. The TLR- or RLH-activated signal is transmitted via the adaptor proteins Toll/IL-1 receptor domain-containing adapter-inducing interferon-β (TRIF)/interferon-β promoter stimulator 1 (IPS-1, also known as MAVS/VISA/Cardif) and leads to the activation of signaling cascades such as NF-κB, interferon regulatory factor 3 (IRF3) and mitogen-activated protein kinase (MAPK). Eventually, IFNs and other proinflammatory cytokines are produced [11].

Recently, protein kinase regulated by dsRNA (PKR), a well-characterized antiviral protein in the IFN system has been indicated as an innate immune regulator [12], [13], [14]. PKR contains two distinct functional regions: two dsRNA binding domains (dsRBDs) at the N-terminus and a kinase domain at the C-terminus [15], [16], [17]. In latent cells, PKR resides in an inactivated form, and is activated by itself or by PKR activating protein (PACT) in response to environmental stresses such as viral infection [18], [19]. Once activated, PKR phosphorylates its substrates including the α subunit of the eukaryotic translation initiation factor 2 (eIF2α) [20], the B56α regulatory subunit of protein phosphatase 2A (PP2A) [21], RNA helicase A (RHA) [22] and Nuclear Factor-90 (NF-90) [23]. By phosphorylating these substrates, PKR plays a role in multiple biological processes including cell growth, transformation, differentiation and apoptosis [24]. For example, the antiviral function of PKR is exerted via phosphorylating eIF2α, hence, attenuating viral protein translation [25], [26]. Overexpression of wild-type PKR, but not the catalytically inactive mutant K296R leads to severe inhibition of cell growth in mammalian, insect and yeast cells, suggesting that PKR controls cell proliferation [27], [28]. Recent evidence has revealed that PKR functions not only as an IFN stimulated gene (ISG) but also as a positive regulator of IFN production in response to viral infection [29]. Furthermore, PKR has been proposed to act as a novel PRR to sense cytoplasmic dsRNA [30], [31]. Several studies have indicated a positive role for PKR in regulating IFNs following infection by vaccinia virus [32], Measles virus [33], West Nile virus (WNV) [30], Semliki Forest virus [34], human parainfluenza virus type 1 [35], lymphocytic choriomeningitis virus (LCMV) [36], and rotavirus [37]. One exception is that the PKR activation is hijacked by Hepatitis C virus (HCV), blocking the IFN production and downstream IFN stimulated gene (ISG) expression [38], [39]. Intriguingly, PKR is also engaged in innate immune responses to non-viral stimuli such as bacterial infection [40], [41], [42], bacterial lipopolysaccharide (LPS) treatment, cytokine challenge or serum deprivation [41], [43], [44].

Notably, the roles played by PKR and the molecular mechanisms through which PKR regulates the innate immune response are quite distinct. For example, PKR is involved in the activation of IRF3 in HeLa cells infected by a vaccinia virus E3L mutant (VVΔE3L) [45] but not by measles virus [33]. The catalytic activity of PKR is required for MAPK activation in VVΔE3L-infected HeLa cells [32] but not in the NF-κB activation in PKR knockout mouse embryo fibroblasts (MEFs) [46], [47]. PKR might regulate IFN production by physically interacting with innate signaling components such as MKK6 [48] and TRAFs [49] or by regulating the IFN mRNA integrity [50]. Although PKR was found to be a negative regulator of chemokine synthesis in mast cells following antibody-enhanced dengue virus infection [51], the role of PKR in DENV infection remains largely unclear.

Herein, we investigated whether PKR is involved in the innate immune response to DENV infection and viral replication by using an established short interfering RNA (siRNA) knockdown technique [33], [52]. Our study utilized a commonly used DENV-permissive cell line, human lung epithelial cells (A549) [53]. Our results demonstrated that PKR played a negative regulatory role in IFN induction. Depletion of PKR significantly upregulated IFN synthesis induced by DENVs or dsRNA. We also showed that PKR downregulated the IFN synthesis via the RIG-I/IPS-1 pathway. The dsRNA binding activity, rather than the catalytic activity of PKR was critical for PKR downregulation of IFN induction. These data suggest that PKR plays a negative regulatory role in the innate immune response and might be a worthwhile target for anti-DENV treatments.

## Materials and Methods

### Reagents

Poly(I:C) was purchased from Sigma (St Louis, MO). Antibodies against PKR, eIF2α, p38, JNK MAPKs,IRF3 and NF-κB p65 were obtained from Santa Cruz (Santa Cruz, CA). Phospho-eIF2α, Phospho-p38, phospho-JNK MAPKs and phospho-IRF3 antibodies were obtained from Cell Signaling Technology (Beverly, MA). The antibody against Phospho-PKR (pT446) was obtained from Epitomics (Epitomics, CA). The β-actin antibody was purchased from Sigma (St Louis, MO).

### Cell Culture

The human lung epithelial cell line A549 (ATCC, CCL-185), the hepatoma cell line HepG2 (ATCC, HB-8065) and the mosquito cell line C6/36 (ATCC, CRL-1660) were maintained in Dulbecco’s modified Eagle’s medium (DMEM) supplemented with 5% or 10% (v/v) fetal bovine serum (FBS) (Gibco,CA), 1% sodium pyruvate, 100 µg/ml of penicillin and 100 units/ml streptomycin (Invitrogen, CA). The human monocytic cell THP-1 (ATCC, TIB-202) was cultured in RPMI-1640 (Gibco,CA) complemented with 10% FBS and 1% 2-mercaptoethanol at 37°C.

### Virus

The Dengue-1 virus Hawaii strain, Dengue-2 virus New Guinea C strain and Dengue-3 virus H241 strain were provided by Guangzhou Centers for Disease Control and propagated in C6/36 cells. C6/36 cells were inoculated with DENV at a low multiplicity of infection (MOI) and incubated at 35°C for 3–7 days. The supernatants were collected and clarified by centrifugation (1000×g, 5 min). Viral concentrations were titered on C6/36 cells, and viral stocks were stored at −80°C.

### Virus Titration

DENV titers in harvested supernatants were determined by TCID50 assay. Samples were serially diluted and added to C6/36 cells in 96-well plates. After a 3–7 day incubation at 35°C and 5% CO_2_, cells were examined for cytopathic effects (CPE) under a light microscope. The virus titer (TCID50/ml) was calculated using the Reed–Muench method. One TCID50/ml was equivalent to 0.69 PFU/ml.

### RNAi

The sequences of chemically synthesized siRNAs were validated by real-time PCR and Western blot. The sequences of siRNAs prepared by Invitrogen with dTdT overhangs were as follows: GCAGGGAGUAGUACUUAAAUA (1) and GCATGGGCCAGAAGGATTTCA (2) for PKR, GGAAGAGGUGCAGUAUAUU for RIG-I, UAGUUGAUCUCGCGGACGA for IPS-1, and GGUGAAGGAGCAGAUUCAG for MDA-5, and Dharmacon’s negative siRNA with a scrambled sequence was used as a control (siNC). siRNAs were transfected into A549 cells by Lipofectamine 2000 (Invitrogen, CA) according to the manufacturer’s instructions.

### Reverse-transcription PCR and Quantitative PCR

Total RNA was prepared from A549 cells using TRIzol reagent (Invitrogen, CA) following the manufacturer’s instructions. Then, 1 µg of total RNA was reverse-transcribed to produce cDNA, and then amplified using SYBR Green Master Mix (Bio-Rad, CA) following the manufacturer’s protocol. The primer pairs used were as follows: (glyceraldehyde-3-phosphate dehydrogenase) GAPDH forward primer GCCTTCCGTGTCCCCACTG and reverse primer CGCCTGCTTCACCACCTTC, IFN-β forward primer AAACTCATGAGCAGTCTGCA and reverse primer AGGAGATCTTCAGTTTCGGAGG, RIG-I forward primer GTTCAGTGAACTGTGGATTGT and reverse primer ACTTCTGAAGGTGGACATGA, and DENV2 C protein forward primer TCCTAACAATCCCACCAACAGCA and reverse primer AGTTCTGCGTCTCCTGTTCAAGA. Quantitative real-time PCR reactions were performed using the CFX96 Real-Time PCR System (Bio-Rad, CA). Relative mRNA levels were calculated after normalization to GAPDH.

### Western Blot

Whole cell extracts were prepared in the presence of 1 mM phenylmethylsulfonyl fluoride (PMSF) and 1% (v/v) protease inhibitor cocktail (Sigma, MO) as described previously (54). Proteins were fractionated by electrophoresis on sodium dodecyl sulfate-10% polyacrylamide gels, transferred to nitrocellulose membranes, blocked, and then probed with the appropriate dilution of primary antibody in phosphate-buffered saline (PBS) containing 3% (w/v) skim milk. Western blot detection was performed with IRDye 800 CW conjugated anti-rabbit IgG or IRDye 680 CW conjugated anti-mouse IgG secondary antibodies according to the manufacturer’s protocols (LI-COR, NE). Immunoreactive bands were visualized using an Odyssey infrared imaging system.

### Indirect Immunofluorescence Microscopy

Cells were seeded onto coverslips (30–50% confluency) and infected with DENV2 (MOI = 1) or transfected with poly(I:C). Cells were fixed at the indicated times by treatments with 4% paraformaldehyde at 4°C for 30 min and permeabilized with PBS containing 0.1% Triton X-100. NF-κB p65 was detected by using a rabbit antibody against p65 (1∶200) and the Alexa Fluor 488-conjugated anti-rabbit antibody (1∶2000, Invitrogen). Stained samples were visualized using a Zeiss fluorescence microscope. Percentage of stained cells was calculated using PicCnt 100x software.

### ELISA

Secreted IFN-β levels in the cell supernatants were determined using a Human Interferon-β ELISA Kit (PBL interferon source, USA) according to the manufacturer’s instructions. Absorbance at 450 nm was read on a microplate reader (Bio-RAD iMark Microplate Reader).

### Statistical Analysis

An unpaired, two-tailed Student’s *t* test was used to determine the significance of real-time PCR and immunofluorescence microscopy data. Data were considered significant at p<0.05.

## Results

### PKR Negatively Regulated DENV-induced IFN Production in A549 Cells

**Figure 1:**
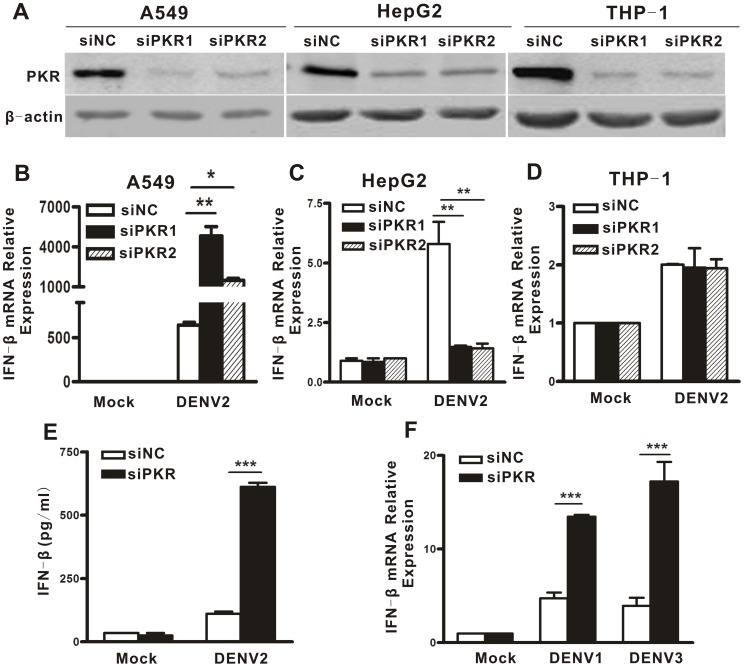
Knockdown of PKR significantly increased IFN-β expression following DENV infection. A549, HepG2 and THP-1 cells were transfected with siRNAs against PKR (siPKR1, and siPKR2) or control siRNA with a scramble sequence (siNC), and harvested at 48 h post transfection for Western blot (A). Transfected cells were infected with DENV2 for 16 h. Expression of IFN-β in DENV2-infected A549, HepG2 and THP-1 cells was evaluated by real-time PCR and normalized to GAPDH (B-D), ELISA was utilized to measure secreted IFN-β in A549 cells following DENV2 infection (E). Expression of IFN-β in DENV1- and DENV3- infected A549 cells was tested by real-time PCR and normalized to GAPDH (F). Data are shown as the mean ± SEM (standard error of the mean) and represent at least three independent experiments. *p<0.05,**p<0.01, ***p<0.001.

We first examined the effect of PKR on IFN induction following DENV2 infection by silencing PKR in three different cell lines: human lung epithelial cells (A549), hepatoma cells (HepG2) and monocytic cells (THP-1). Western blot data showed that transfection of two different siRNAs against PKR (siPKR1 and siPKR2) resulted in a greater than 70% reduction of PKR expression in these cells (Figure 1A), validating the efficacy of the siPKRs. Next, IFN-β expression in PKR sufficient and deficient cells following DENV2 infection was measured by real-time PCR. PKR depletion had differential effects on IFN expression in A549, HepG2 and THP-1 cells infected by DENV2. PKR knockdown significantly increased expression levels of IFN-β in A549 cells (Figure 1B, black and hatched bars vs white bar, p = 0.0048 and p = 0.0148), while IFN-β expression levels in DENV2-infected HepG2 cells was reduced by PKR knockdown (Figure 1C, black and hatched bars vs white bar, p = 0.095 and p = 0.0088). With THP-1 cells PKR silencing had no significant effect on IFN-β levels (Figure 1D). Secreted IFN-β protein, in the DENV-infected A549 cells was further measured by ELISA. As shown in Figure 1E, the PKR depletion significantly enhanced the secreted IFN-β amount in response to DENV2 infection (black bars, p<0.0001), in agreement with the above real-time PCR data.

Next, the effects of PKR on the IFN-β production induced by infection by two other DENV serotypes were examined. The real-time PCR data showed that PKR depletion increased IFN-β expression by 3- and 4.6-fold in DENV1 and DENV3 infected A549 cells respectively (Figure 1F, p = 0.0008 and p<0.0001), suggesting that the regulatory effect of PKR on the DENV-induced IFN production was not serotype specific.

### PKR Downregulated the Activation of the MAPKs p38 and JNK, IRF3 and NF-κB

**Figure 2:**
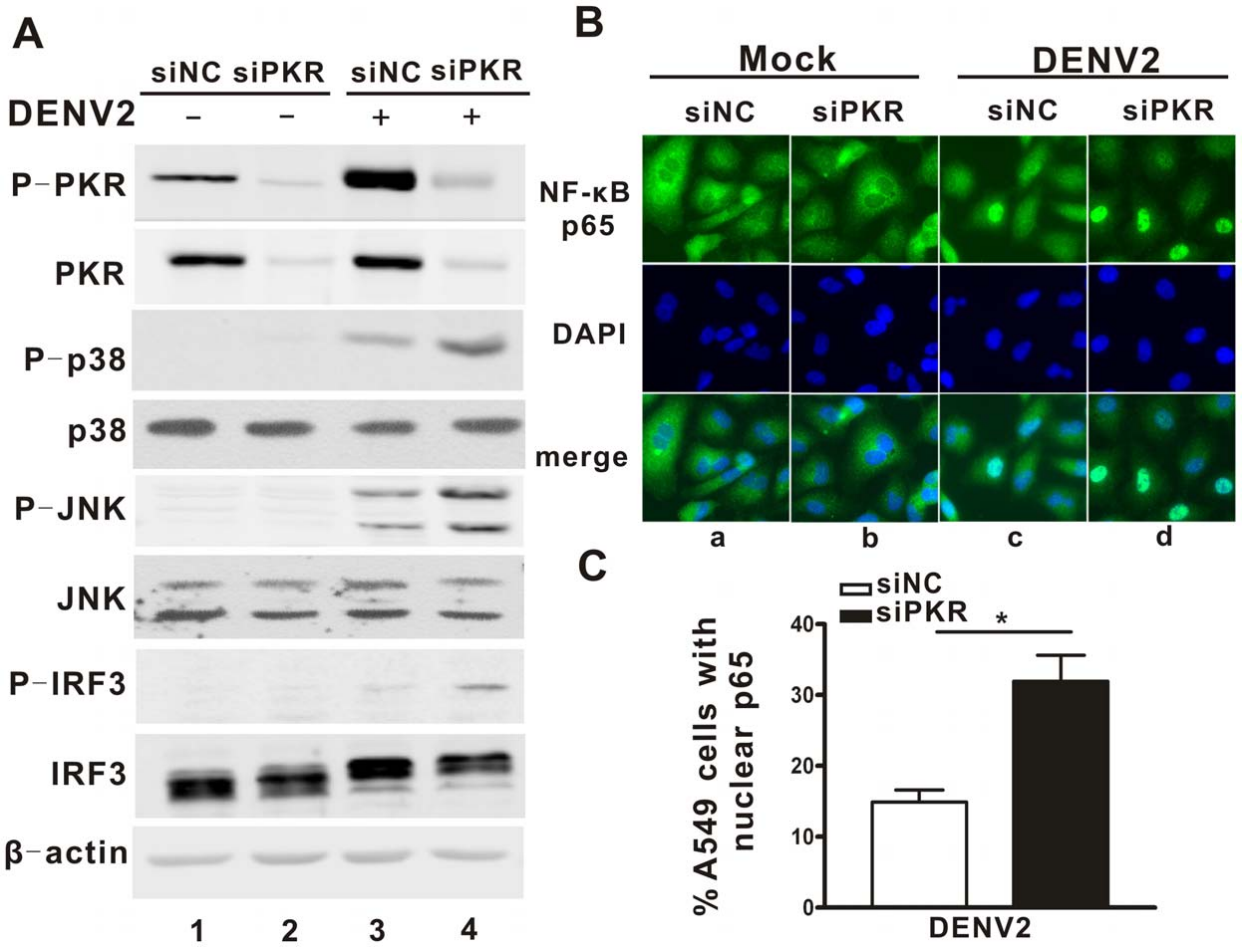
PKR silencing enhanced the activation of p38 and JNK MAPKs, IRF3 and NF-κB. A549 cells were transfected with siPKR1 or siNC followed by infection with DENV2. (A), Western blot to show the phosphorylation form and total protein levels of **the** MAPKs p38 and JNK, IRF3 and PKR. (B), Immunoflurescence microscopy images showing the subcellular localization of NF-κB p65. (C), PicCnt 100x was used to determine the percentages of cells with nuclear staining for NF-κB. Data are shown as the mean ± SEM and represent at least three independent experiments. *p<0.05.

Knockdown of PKR by both siRNAs resulted in an elevated IFN induction following DENV2 infection, though to varied extents, which could be influenced by differential knockdown efficacies. To further investigate the underlying mechanisms, siPKR1, the more efficient siRNA was utilized in subsequent experiments. First, we examined the activation of three important signaling cascades involved in the transcriptional regulation of IFN-β, including MAPKs (p38 and JNK), IRF3 and NF-κB in the PKR sufficient and deficient cells. After DENV2 challenge, the PKR phosphorylation levels were significantly increased in PKR sufficient cells (Figure 2A, *lane* 3 vs *lane* 1), while both of total PKR and the phosphorylated form were largely abolished by PKR silencing (Figure 2A, *lanes* 2 and 4). Next, activation of MAPKs and IRF3 was examined by Western blot using antibodies specific for their phosphorylated forms. Increased phosphorylation levels of p38, JNK and IRF3, were observed in all cells infected by DENV2 (Figure 2A, *lanes* 3, 4 vs *lanes* 1, 2). In addition, the phosphorylation levels of p38, JNK and IRF3 in PKR knockdown cells (Figure 2A, *lane* 4) were elevated further than in PKR sufficient cells (Figure 2A, *lane* 3). As a control, the total protein amounts of p38, JNK and IRF3 were comparable in all the cells examined.

As NF-κB is released from IκB and translocated into the nucleus upon activation, we monitored the NF-κB activation by examining its subcellular distribution by indirect immunofluorescence microscopy. In mock-infected A549 cells, the NF-κB resided predominantly in the cytoplasm, as expected (Figure 2B, *panels* a and b). In response to DENV2 infection, NF-κB protein was translocated into the nucleus (Figure 2B, *panels* c and d). However, the percentages of nuclei positively staining cell nuclei were significantly higher in the PKR deficient cells with the PKR sufficient cells (30% vs 15% in Figure 2B *panel* d vs *panel* c, respectively; Figure 2C, p = 0.0139). These data suggested that PKR had a negative impact on the MAPKs, IRF3 and NF-κB signaling cascades triggered by DENV2 infection.

### PKR Downregulation of IFN Induction was Dependent on RIG-I and IPS-1 but not MDA-5

**Figure 3:**
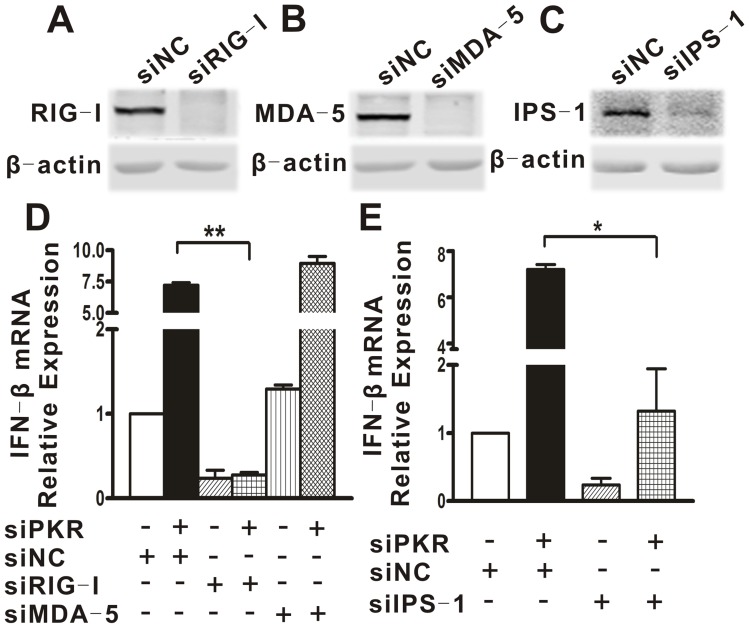
The IFN induction mediated PKR knockdown was dependent on RIG-I and IPS-1, but not MDA-5. A549 cells were transfected with siRNA against RIG-I (siRIG-I), MDA-5 (siMDA-5) or IPS-1 (siIPS-1). Protein levels of RIG-I (A), MDA-5 (B) and IPS-I (C) were detected by Western blot. A549 cells were cotransfected with siRIG-I, siMDA-5 or siIPS-1 together with siPKR1 or siNC for 48 h followed by DENV2 infection (D, E). Total cellular RNA was analyzed for IFN-β using real-time PCR and normalized to that of GAPDH in each sample. Data are shown as the mean ± SEM and represent at least three independent experiments. *p<0.05, **p<0.01.

To further probe how PKR modulates the IFN production and transcription factor activation, we examined the relationship between PKR and three upstream innate immune-related proteins: PRRs (RIG-I and MDA-5) and their adaptor IPS-1. siRNA against RIG-I (siRIG-I), MDA-5 (siMDA-5) or IPS-1 (siIPS-1) was co-transfected together with siPKR1 or siNC into A549 cells, followed by DENV2 challenge. Transfection of siRIG-I, siMDA-5 or siIPS-1 resulted in significant repression of the corresponding protein in A549 cells, validating the efficacy of these siRNAs (Figure 3A, 3B and 3C). The real-time PCR analysis showed that depletion of RIG-I dramatically decreased DENV2-induced IFN expression in both PKR sufficient and deficient cells (Figure 3D, p = 0.001), indicating that RIG-I is important for innate immune signal transduction in PKR sufficient cells, and also is indispensable for the IFN induction mediated by PKR knockdown. By contrast, the MDA-5 silencing did not change the IFN levels in either PKR sufficient or deficient cells infected by DENV2 (Figure 3D). In addition, silencing of the adaptor of RIG-I and MDA-5, IPS-1, also largely abolished DENV2-induced IFN-β expression (Figure 3E, p = 0.012). These data suggested that the regulatory effect of PKR on IFN production was dependent on the RIG-I and IPS-1.

### The dsRNA Binding Activity of PKR was Required for its Regulatory Effect on IFN

**Figure 4:**
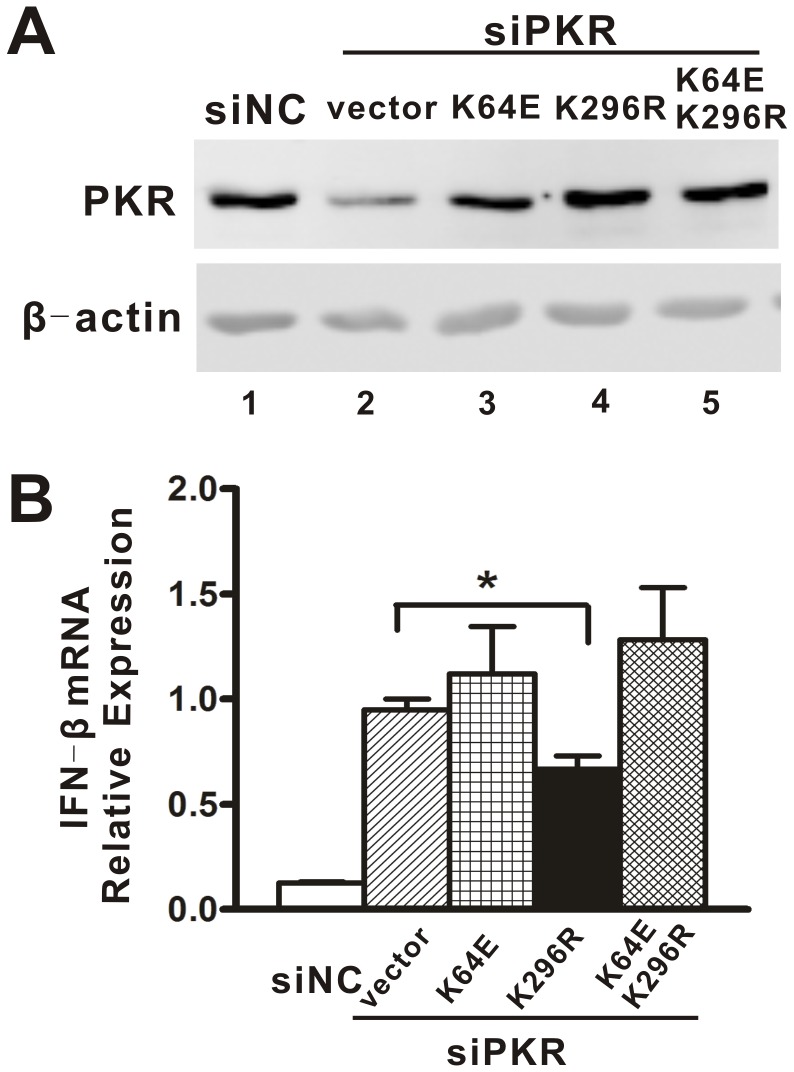
The dsRNA binding activity of PKR was important for downregulation of IFN induction. A549 cells were transfected with pcDNA6, pcDNA6-K64E, pcDNA6-K296R or pcDNA6-K64EK296R followed by siNC or siPKR1 transfection. Cells were then harvested for Western blot analysis (A). A549 cells transfected with different PKR mutants and siPKR1 were stimulated by DENV2 infection. Total cellular RNA was harvested for real-time PCR analysis (B). Data are shown as the mean ± SEM and represent for at least three independent experiments. *p<0.05.

To separate the potential role of the two main PKR functions in PKR regulation of IFN production, its dsRNA binding activity and its catalytic activity in the PKR regulation of IFN production, we expressed three different PKR mutants: the K64E mutant which lacks most dsRNA binding activity, the K296R mutant which lacks catalytic activity, and the double mutant K64EK296R which lacks both activities. Endogenous PKR was then silenced by RNAi, after which the cells were infected by DENV2. To circumvent the knockdown of the plasmids in the transfected cells by siPKR1, the three PKR expression plasmids were mutated at the positions targeted by the silencing RNA without altering the PKR amino acid sequence. Western blot analysis showed that the PKR expression levels in these cells (Figure 4A, *lanes* 3, 4, 5) were comparable to the levels of endogenous PKR in wild type A549 cells (Figure 4A, *lane* 1), while significantly lower PKR expression was observed in the cells transfected with the vector plasmid and siPKR1 (Figure 4A, *lane* 2). Transfection of the vector plasmid in PKR knockdown cells led to a significantly higher expression of IFN-β in those cells (Figure 4B, hatched bars) than in siNC-transfected cells (Figure 4B, white bars) following DENV2 infection. Complementation with K296R protein significantly reversed the PKR-knockdown-mediated IFN-β induction by 35% (Figure 4B, black bars) compared with vector-transfected cells (Figure 4B, hatched bars) (p = 0.036). By contrast, the expression of two other mutant proteins, the K64E or the K64EK296R mutants, had no or minimal effect on the IFN production. These results indicated that the dsRNA binding activity of PKR, but not its catalytic activity, is important for PKR to negatively regulate the IFN production.

### PKR Knockdown did not Alter DENV2 Replication in A549 Cells

**Figure 5:**
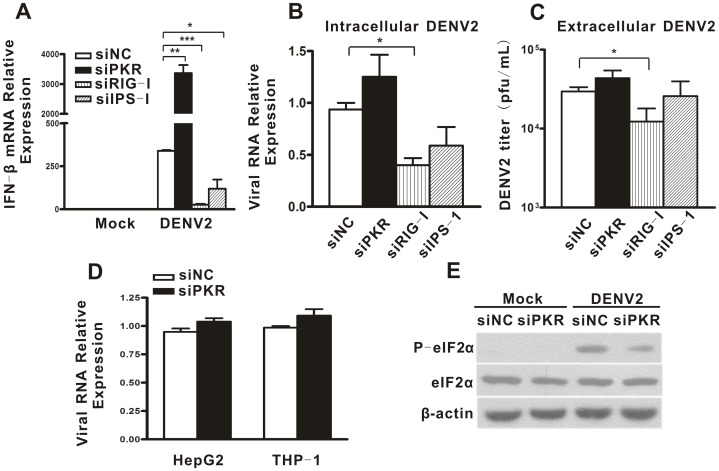
PKR depletion barely affected DENV2 replication in A549 cells. A549 cells were transfected with siNC, siPKR1, siRIG-I and siIPS-1, followed by DENV2 infection. Cells and culture supernatants were harvested for real-time PCR and viral titration respectively. Total cellular RNA was analyzed for IFN-β (A) or DENV2 RNA (B) level by real-time PCR and normalized to that of GAPDH in each sample. Virus yields were determined by TCID50 assay (C). DENV2 RNA levels in siNC- or siPKR-transfected HepG2 and THP-1 cells were measured by real-time PCR (D). Data are shown as mean ± SEM at least three independent experiments. Phosphorylation form and total protein levels of eIF-2α in DENV2-infected A549 cells were detected by Western blot (E). *p<0.05,**p<0.01, ***p<0.001.

To explore whether the negative regulation of PKR on IFN-β has a direct impact on the viral replication, we compared DENV2 RNA levels and extracellular yields by real-time PCR and a TCID50 assay. The real-time PCR data revealed that high levels of DENV2 RNA were detected in the A549, HepG2 and THP-1 cells (Table 1), validating that these cell lines are permissive to DENV replication. Next, A549 cells were transfected with siPKR1, siRIG-I and siIPS-1, followed by DENV2 infection. Infected cells and cell supernatants were harvested at 16 h post infection for analysis of viral RNA levels and viral yields. As expected, PKR knockdown enhanced IFN-β production while knockdown of RIG-I or IPS-1 decreased IFN-β production (Figure 5A, p = 0.0077, p = 0.0005 and p = 0.0488). Both of the viral RNA levels (Figure 5B, p = 0.0279) and viral yields (Figure 5C, p = 0.0483) in PKR or IPS-1 knockdown cells were comparable to the control cells. Surprisingly, silencing of RIG-I and IPS-1 did not enhance the viral RNA level and viral yield (Figure 5B and 5C), although their absence alleviated the IFN production (Figure 5A). Moreover, PKR silencing in HepG2 and THP-1 cells did not have a significant effect on the viral RNA level (Figure 5D).

**Table 1:** Viral RNA levels in DENV2-infected A549, HepG2 and THP-1 cells.

Cells	Sample	Ct value for Viral RNA	Ct value for GAPDH
**A549**	MockDENV2	ND14.28±0.42	19.76±0.3719.30±0.49
**HepG2**	MockDENV2	ND21.46±0.60	17.68±0.4517.47±0.26
**THP-1**	MockDENV2	ND23.85±0.72	18.28±0.5818.68±0.47

DENV2 RNA levels in A549, HepG2 and THP-1 cells were measured by real-time PCR and the values of cycle threshold (Ct) of viral RNA and GAPDH were analyzed. ND, not detected.

To probe the possible reason why higher levels of IFN-β in PKR knockdown A549 cells were not correlated with inhibition of DENV replication, we further examined the activation level of the PKR substrate eIF-2α, which inhibits protein translation when it is phosphorylated. As shown in Figure 5E, the phosphorylated form of eIF-2α was readily detected in DENV-infected control cells, but PKR knockdown significantly abolished eIF-2α phosphorylation. As a control, the total eIF-2α amounts were shown to be comparable in all tested cells. These data suggested that PKR knockdown did not influence DENV2 replication in A549 cells possibly due to its two opposing effects, namely, the enhancement of IFN-β and the impairment of eIF-2α activation.

### PKR Inhibited Poly(I:C)-mediated IFN Induction in A549 Cells

**Figure 6:**
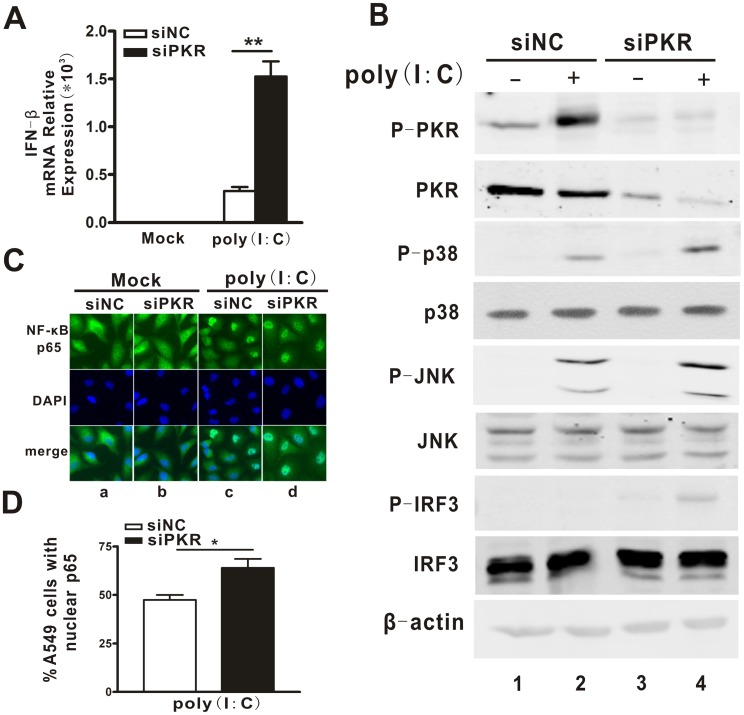
PKR knockdown enhanced IFN expression and activation of MAPKs, IRF3 and NF-κB pathways induced by poly(I:C). A549 cells were transfected with siPKR1 or siNC for 48 h, and then transfected with poly(I:C) for 1 h. Expression levels of IFN-β (A) were tested by real-time PCR and normalized to GAPDH. Phosphorylation form and total protein of p38 and JNK MAPKs, IRF3 and PKR were detected by Western blot (B). Immunoflurescence microscopy images to show subcellular localization of NF-κB p65 (C). PicCnt 100x was used to determine the percentages of cells stained with nuclei NF-κB (D). Data are shown as mean ± SEM for at least three independent experiments. *p<0.05,**p<0.01.

To answer the question of whether the negative regulatory effect of PKR on IFN induction in A549 cells is DENV-specific, the synthetic dsRNA analogue poly(I:C) was used for further studies. In response to poly(I:C) transfection, expression of IFN-β in the PKR-silenced cells (Figure 6A, black bar) was significantly more elevated (by 5-fold) than in the PKR control cells (Figure 6A, white bar, p = 0.0019), indicating that the regulatory role of PKR in IFN production is at the level of the dsRNA.

Next, the effects of PKR knockdown on the activation extent of the p38 and JNK MAPKs, and IRF3 by poly(I:C) were examined. As shown in Figure 6B, the phosphorylation of p38, JNK and IRF3 in the PKR knockdown cells was significantly higher than that in the control cells following poly(I:C) transfection, while the total amounts for the p38 and JNK MAPKs, and IRF3 proteins were comparable between the PKR knockdown cells and control cells. Moreover, indirect immunofluorescence microscopy results showed that the percentage of cells with nuclear translocation of NF-κB was significantly higher in PKR deficient cells than in PKR sufficient cells (Figure 6C and 6D, p = 0.0198), suggesting that PKR negatively regulates dsRNA-induced activation of the MAPKs, IRF3 and NF-κB pathways in A549 cells.

**Figure 7:**
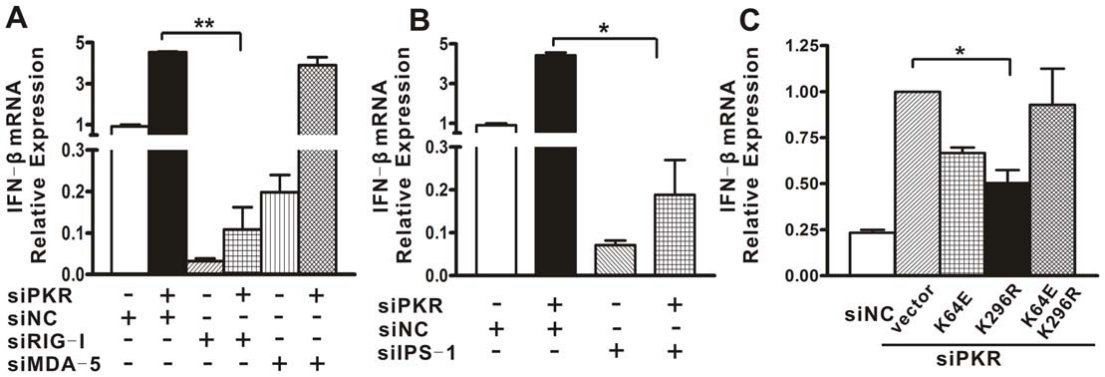
Suppression of poly(I:C) induced IFN by PKR required RIG-I/IPS-I and dsRNA binding activity of PKR. (A, B) A549 cells were co-transfected with siRIG-I, siMDA-5 or siIPS-1 together with siPKR1 or siNC followed by poly(I:C). Total cellular RNA levels were analyzed for IFN-β using real-time PCR and normalized to those of GAPDH in each sample. (C) Cells were transfected with pcDNA6, pcDNA6-K64E, pcDNA6-K296R or pcDNA6-K64EK296R followed by siNC or siPKR1 transfection, and were then stimulated by poly(I:C). Total cellular RNA was analyzed for IFN-β expression by real-time PCR and normalized to that of GAPDH in each sample. Data are shown as mean ± SEM at least three independent experiments. *p<0.05, **p<0.01.

We then tested the role of RIG-I, MDA-5 and IPS-1 in the PKR-mediated IFN expression by co-transfecting their specific siRNA. The real-time PCR data showed that regardless of the PKR status, knockdown of RIG-I or IPS-1 but not MDA-5, significantly inhibited the IFN induction following poly(I:C) treatment (Figure 7A and 7B, p = 0.0002, p = 0.0013), suggesting that the regulatory effect of PKR on poly(I:C)-induced IFN also relied on RIG-I/IPS-1. Finally, we examined the requirement of the dsRNA binding activity and kinase activity for the PKR regulatory activity. The PKR mutants, K64E, K296R and K64EK296R were overexpressed in cells followed by transfection with siPKR1 and poly(I:C). Complementation with the K296R protein (Figure 7C, p = 0.02), but not the other two mutants, significantly alleviated the enhancement mediated by PKR knockdown, consistent with the data obtained with DENV2 infection.

### RIG-I was Differentially Expressed in A549, HepG2 and THP-1 Cells

**Figure 8:**
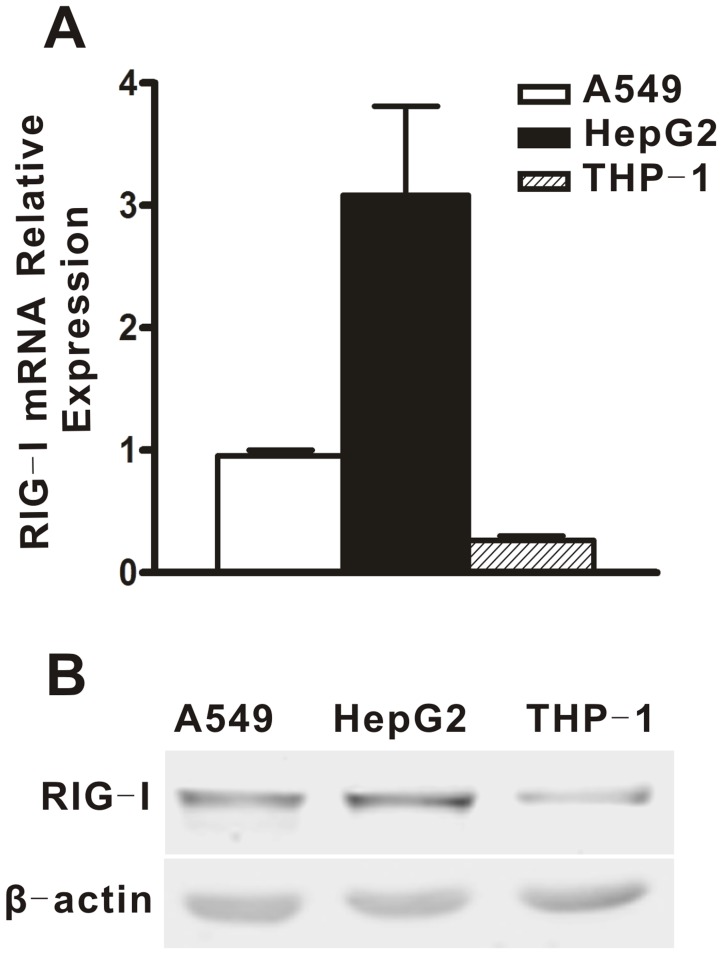
Expression of RIG-I in different cell lines. A549, HepG2 and THP-1 cells were harvested for real-time PCR analysis (A) or for Western blot analysis (B). Total cellular RNA was analyzed for RIG-I expression by real-time PCR and normalized to that of GAPDH in each sample. Data are shown as mean ± SEM at least three independent experiments.

In response to both DENV and poly(I:C), RIG-I was found to be critical for PKR to negatively regulate IFN-β induction. Thus, we compared endogenous expression of RIG-I in A549, HepG2 and THP-1 cells to explore the possibility that RIG-I is a factor involved in the differential effects of PKR on the IFN-β induction in these different cell types. RIG-I mRNA and protein levels were determined by real-time PCR and Western blot. In Figure 8A and 8B, the RIG-I expression in A549 cells was approximately 3-fold less than in HepG2 cells. In THP-1 cells, the mRNA and protein levels of RIG-I were much lower than in the other two cell types. These data revealed that different levels of endogenous RIG-I were present in A549, HepG2 and THP-1 cells.

## Discussion

PKR, an IFN-inducible antiviral protein, has recently come into focus as a regulator of innate immunity [30]. The significance of PKR in a variety of biological processes prompted us to test its involvement in the IFN production in response to DENV infection. Our work demonstrated that PKR negatively regulated IFN expression in A549 cells following either infection of DENVs or treatment with dsRNA. This negative regulatory role of PKR in IFN production was dependent on RIG-I/IPS-1, and their downstream signaling pathways. We also demonstrated that the dsRNA binding activity of PKR was important for its activity to inhibit IFN production.

Our first finding was that PKR negatively regulated the IFN expression in A549 cells in response to the stimulation by either DENV infection or poly(I:C) treatment. This observation is in contrast to previous reports that PKR knockout, PKR knockdown or PKR inhibition lead to a reduction in virus-triggered IFN production [30], [32], [33], [34], [35], [36], [37]. We postulated that the negative regulatory activity of PKR in innate immunity is dependent on the cell type, rather than on the virus type based on the following observations. Firstly, PKR plays a positive or negligible role in IFN production in other DENV-permissive cell lines, such as HepG2 and THP-1 cells. Secondly, IFN production was also upregulated in PKR knockdown A549 cells when stimulated by poly(I:C), which represents a common byproduct of many viruses [54]. Furthermore, we consistently observed that a higher IFN induction level by other TLR ligands could be more-readily achieved in A549 cells than in THP-1 or HepG2 cells (data not shown). The potent IFN producing ability of epithelial derived A549 cells might be attributed to its higher abundance of certain innate immune related proteins. The finding that PKR plays different regulatory roles in different cells might reflect a flexible strategy by PKR to protect cells. In potent IFN producing cells, PKR inhibits the induction of IFNs to prevent over-responsiveness, while in other cells PKR boosts the induction of IFNs to build a robust antiviral state.

The activation of MAPKs, IRF-3 and NF-κB is known to be required for the production of IFN [55]. Our data revealed that in response to DENV2 or poly(I:C) stimulation, PKR knockdown promoted the phosphorylation levels of p38, JNK and IRF-3, as well as the nuclear translocation of NF-κB, implying that PKR regulates the activation pathway of p38, JNK, IRF-3 and NF-κB. Interestingly, the mechanism through which PKR negatively regulates IFN production is similar to its positive regulatory mechanism in other models, namely through modulating the activation of signaling cascades. As PKR has a broad effect on all activation cascades of MAPKs, IRF3 and NF-κB, we presumed that PKR might act at a common upstream point, such as the level of PRRs or adaptor proteins. Because TLR3 is not found on the membrane of A549 cells [56], the cytoplasmic PRRs that recognize DENV2 and poly(I:C), RIG-I, MDA-5, and the adaptor IPS-1, were tested in this study. Our work revealed that depletion of RIG-I or IPS-1 abrogated the IFN induction mediated by PKR knockdown, indicating that RIG-I is the dominant PRR in sensing DENV2 and poly(I:C) in A549 cells and is involved in the downregulatory effects of PKR on IFN production. However, the depletion of MDA-5 had a marginal impact on the DENV2 induced IFN level regardless of the PKR status, suggesting that in A549 cells, MDA-5 signaling may be compensated for other redundant pathways such as RIG-I.

Our work demonstrated that the dsRNA binding activity of PKR rather than its catalytic activity is required for its downregulation of IFN induction. Overexpression of the PKR catalytically inactive PKR mutant K296R, but not the K64E mutant or K64EK296R double mutant was able to reverse the effect induced by PKR knockdown. Based on this observation and other literature [57], we propose that PKR might serve as a dsRNA binding protein, rather than as a kinase, to downregulate the innate immune signaling. In addition to PKR, many other innate-immunity related proteins, including RIG-I, MDA-5 and PACT, are activated by dsRNA. Specifically, our data verified that RIG-I levels in the A549 cells were much lower than in the HepG2 cells. Therefore in wild type A549 cells, PKR might compete with RIG-I for dsRNA binding in two possible ways: first by binding dsRNA ahead of RIG-I, and second because PKR would be more abundant than RIG-I in the A549 cells. As a consequence of PKR depletion, dsRNA becomes more available for RIG-I binding and activation, leading to a stronger innate immune response. In HepG2 cells, PKR competition for dsRNA binding might be overwhelmed by relatively more-abundant RIG-I protein, so the activation of RIG-I could reach a high enough threshold to initiate the IFN signaling. PKR might further promote the IFN induction by interacting with TRAF-6 [49] such that its depletion disrupts their interaction and the subsequent decrease of IFN induction. Given the low IFN induction levels in THP-1 cells challenged by DENV2 or poly(I:C), and the substantially lower expression of RIG-I in THP-1 cells, we deduced that the low abundance or lack of RIG-I or other factors might limit the innate immune signaling transduction, even in the context of increasing dsRNA in the absence of PKR. Therefore, the presence or absence of PKR did not show a differential effect on the IFN synthesis in challenged THP-1 cells. Our proposed dsRNA-sequestering mechanism of PKR is similar to that of LGP2, a third RLR family member, which prevents the RIG-I and MDA-5 recognition of dsRNA [58]. Our hypothesis is supported by a recent report by the Meurs group that PKR is able to bind HCV RNA ahead of RIG-I [57]. Undoubtedly, different impacts of PKR on IFN induction in different cells can result from complex interactions between several factors, of which RIG-I is a critical but not exclusive factor involved. For example, the involvement of PACT cannot be ruled out. PACT activation might also be dampened by insufficient levels of dsRNA molecules due to PKR competition. Moreover, as an activator of both RIG-I and PKR, PACT could be occupied by PKR interactions in PKR sufficient cells so that RIG-I could not form a complex with PACT and therefore become activated [59]. However, in PKR deficient cells, PACT can be activated by dsRNA and interact with RIG-I to initiate signaling.

Although PKR has previously been reported to downregulate the IFN production following HCV infection, the underlying mechanism apparently differs from that in our model. In the HCV model, the catalytic activity of PKR was required to phosphorylate eIF2α and selectively inhibited the translation of host cellular proteins, including IFNs [38], [39]. In contrast, in the present work, the regulatory effects of PKR were independent of its kinase activity and instead dependent on its dsRNA binding activity.

Interestingly, we found that the enhanced IFN production induced by PKR depletion did not lead to a lower replication levels of DENV2 in A549 cells. One possibility is that there might be a balance between the two aspects of PKR depletion. PKR knockdown not only enhanced IFN induction but also impaired eIF-2α phosphorylation, thus potentially increasing the efficiency of viral protein translation. On the other hand, because of the timing of IFN secretion, the IFN levels triggered by DENV infection might not be correlated with DENV replication. Pretreatment with IFNs protects cells from DENV infection, but treatment with IFNs following DENV infection does not have this protective effect [60] due to inhibition of the IFN signaling pathway by viral NS2A, NS4A, NS4B and NS5 [61], [62]. This hypothesis was also supported by our observation that DENV replication was not correspondingly enhanced in RIG-I and IPS-1 knockdown cells. Moreover, the possibility that the infected A549 cells might not be responsive to the IFNs they secreted could not be ruled out. Given these possibilities, it is not surprising that PKR knockdown barely affected DENV replication, consistent with the Harris group’s report that the inhibition of DENV replication mediated by IFN-β is not affected in the PKR null MEFs [8]. Interestingly, in our cells, RIG-I silencing inhibited viral replication, while in another model, RIG-I knockdown in HUH-7 cells enhanced DENV1 replication [63]. These contradictory observations regarding the role of RIG-I in DENV replication could be a viral- or cell-specific effect and needs to be further investigated.

In summary, our study presents evidence that PKR downregulates the induction of IFN in response to DENVs or poly(I:C) in a cell-specific manner by controlling the activation of MAPKs, IRF-3 and NF-κB. This negative regulatory role of PKR requires the participation of RIG-I and IPS-1. Our findings expand the PKR functions to that of a negative regulator of the innate immune response, and highlight the importance of PKR as a sentinel in the anti-DENV defense.
